# Hip displacement management in spinal muscular atrophy in the era of disease modifying therapies: a Delphi consensus study in the UK

**DOI:** 10.1016/j.eclinm.2026.103872

**Published:** 2026-04-18

**Authors:** Maria I. Vanegas, Giovanni Baranello, Fabian Norman-Taylor, Michail Kokkinakis, The UK SMA Hip Consensus Group, M. Kokkinakis, M. Vanegas, J. Sheehan, E. Wraige, G. Baranello, F. Norman-Taylor, A. Manzur, M. Scoto, A. Hipwell, E. O'Reilly, M. Main J Longatto, R Muni Lofra, M. Mifsud, R. Buckingham, F. Henderson, S. Ramdas, M. Illingworth, M. Geary, I. Hughes, I. Shah, S. Warner, N. Emery, N. Kiely, T. Willis, J. Cashman, M. Ong, K. White, J. Turner, N. Taylor, A. Majumdar, W. Atherton, C. Edwards, C. Frimpong-Ansah, I. Horrocks, C. Murnaghan, S. McKenzie, R. Keetley, E. Dowling, L. Pallant, A. Fishlock, A. Childs, J. Widnall, R. Madhu, S. Gregson, D. Baskaran, Z. Lambat, S. Annamalai, J. Taylor, C. Carpenter, R. Rabb, A. Cosgrove, G. Nicfhirleinn, P. Thorman, M. Carsi

**Affiliations:** a Evelina London Children's Hospital, Guy's & St Thomas' NHS Trust, London, UK; b The Dubowitz Neuromuscular Centre, Developmental Neurosciences Research and Teaching Department, UCL Great Ormond Street Institute of Child Health, National Institute for Health Research Great Ormond Street Hospital Biomedical Research London, UK; c Great Ormond Street Hospital for Children NHS Foundation Trust, London, UK; d King's College London, London, UK

**Keywords:** Spinal muscular atrophy, Hip displacement, Orthopaedic management, Disease-modifying therapy, Delphi consensus

## Abstract

**Background:**

Spinal muscular atrophy (SMA) is a genetic neuromuscular disorder caused by bi-allelic deletions or pathogenic variants in the *SMN1* gene. SMA type 1 is the most severe form with early muscle weakness, failure to achieve motor milestones, and limited survival. Disease-modifying therapies (DMT) nusinersen, risdiplam, and onasemnogene abeparvovec, have improved survival and motor outcomes but have also created new challenges, including more complex orthopaedic care. Management of hip displacement in symptomatic children with SMA remains controversial, with approaches ranging from conservative to surgical. We aimed to conduct a Delphi consensus exercise in the United Kingdom (UK) to provide national guidance.

**Methods:**

This Delphi consensus process began in September 2023 and included two rounds involving 45 senior health care professionals (paediatric neurologists, orthopaedic surgeons, physiotherapists) and patient representatives from 19 leading paediatric neuromuscular centres in the UK. The consensus process focussed on the prevention and management of hip displacement in children with SMA, supported by the SMA REACH and SMA CARE Networks, the British Society for Surgery in Cerebral Palsy (BSSCP), and British Society for Children's Orthopaedic Surgery (BSCOS). Round 1 was performed online (August–September 2024) and included a questionnaire of 16 statements. The questionnaire was distributed through members of the SMA REACH Network and members of BSCOS and BSSCP. A representative member from the advocacy group SMA UK was also invited to participate. Round 2 was performed in a hybrid manner (combined online and in-person participation) and allowed for live voting, modification, and final approval of approved statements. Input from patient representatives also informed the discussion.

**Findings:**

Of the 23 paediatric neuromuscular centres invited to participate, 19 centres agreed. Round 1 included 44 respondents voting on 16 statements, resulting in consensus (>75% agreement) on six and rejection of three statements. Seven were included for Round 2 discussion. Following live voting among 45 respondents in Round 2, the final consensus included 13 approved statements addressing key aspects of hip and contractures management in SMA. The recommendations emphasise individualised, multidisciplinary assessments and proactive strategies to prevent hip dislocation, particularly in children with higher motor potential, while acknowledging the lack of current evidence and the need to collect long-term data. Key recommendations included timeline for radiographic hip surveillance, and orthopaedic approach to painful hips as well as muscle and joint contractures. The consensus highlights the importance of building upon the existing national database (SMA Reach UK registry) and of developing evidence-based guidelines for both conservative and surgical approaches. The potential role of less invasive approaches was discussed as an option for selected cases.

**Interpretation:**

This study emphasises the importance of multidisciplinary collaboration and individualised care in optimising orthopaedic management for patients with SMA. By addressing gaps in clinical practice, the consensus recommendations provide a foundation for consistent, evidence-based care while promoting research and audit initiatives. This is the current evidence and clinical expertise based on national guidance for the UK and the first of its kind internationally. Future multicentre prospective studies and standardised registries are needed to evaluate long-term clinical, functional, and surgical outcomes and to develop evidence-based guidance for hip management in children with SMA.

**Funding:**

Novartis, Roche, and Biogen.

**Research in context**

**Evidence before this study**

Hip displacement is a common finding in patients with Spinal Muscular Atrophy (SMA), and with the evolving phenotypes after disease-modifying therapies (DMTs), particularly in children with SMA 1 treated after symptom onset, there is controversy on how to prevent or manage hip dislocation.

We conducted a systematic literature search with no language restrictions on PubMed, from database inception to September 2025. Search terms included “hip surgery in SMA,” “hip displacement in SMA,” “pain hips SMA,” and “orthopaedic surgery in SMA.”

Twelve studies were considered that highlighted a more proactive approach in recent years, suggesting to treat hip instability to capitalize on the treatment's effects and allow for maximal mobility.

**Added value of this study**

The present study is a first national initiative in the UK that attempts through a Delphi consensus to provide some guidance for the surveillance and management of hip displacement in treated symptomatic children with SMA.

Agreement was achieved on 13 statements that outline recommendations to early detect and manage hip displacement in the current rapidly changing clinical landscape.

**Implications of all the available evidence**

A multidisciplinary team approach to manage musculoskeletal problems in children with SMA will be essential to maximise standards of care and the effects of DMTs. Future work will require longitudinal data collection and integration into national registries to identify benefits of a more pro-active monitoring and management of hip displacement in children with SMA.

## Introduction

Spinal muscular atrophy (SMA) is an autosomal recessive disorder secondary to bi-allelic deletions or pathogenic variants in the Survival Motor Neuron (*SMN1*) gene. Lack of survival motor neuron (SMN) protein due to *SMN1* gene defect leads to motor neuron loss and progressive muscle weakness. SMA has historically been classified into four types based on the severity of presentation.^1^ Type 1 SMA, the most severe form, is characterized by early-onset muscle weakness, failure to achieve motor milestones, and limited survival beyond two years without ventilatory support.^2^ The introduction of disease-modifying therapies (DMTs) has significantly changed the landscape, improving survival rates and motor development.^3^

Currently, there are three approved DMTs for SMA. Nusinersen (SPINRAZA®), an intrathecal antisense oligonucleotide, targeting *SMN2*; Risdiplam (EVRYSDI®), an oral *SMN2* pre-mRNA splicing modifier; and Onasemnogene abeparvovec (ZOLGENSMA®), a gene replacement therapy using adeno-associated virus 9 (AAV9). Early initiation of a DMT, especially when started pre-symptomatically, can significantly alter the course of the disease, resulting in unprecedented outcomes, with most patients achieving developmental milestones and avoiding the need for respiratory or feeding support.^4^

Improved survival rates have led to new phenotypes and challenges in multidisciplinary management, including orthopaedic care, particularly when patients are treated after the onset of symptoms.^5^^,^^6^ One major change is the acquisition of motor milestones in treated SMA 1 patients. Real-world data show that up to 76·5% treated patients gain head control, up to 66·7% achieve independent sitting, up to 35·3% and to 7·8% can stand with and without support, respectively.^6^^,^^7^ These new functional phenotypes, such as independent sitters, standers, and walkers, offer a more accurate classification of SMA based on the current motor milestone achieved, and are better suited to guide treatment decisions, including the consideration of surgical interventions for musculoskeletal issues previously ignored.^8^

Musculoskeletal complications in children with SMA include hip displacement, scoliosis, and muscle and joint contractures.^9^ Hip dislocation has an early onset in SMA with a reported prevalence/age of onset (mean, years) of 84%/3·1 yrs in type SMA type 1, 80%/5·8 yrs in type SMA type 2, and 36%/9 yrs in SMA type 3 respectively.^10^, ^11^, ^12^, ^13^ As survival rates improve, the number of SMA type 1 patients seeking care for their musculoskeletal conditions is expected to rise, particularly in countries where newborn screening for SMA is not widely implemented, making early monitoring and intervention crucial.^12^, ^13^, ^14^

Studies comparing SMA with Cerebral Palsy (CP) have shown some similarities in proximal femoral changes, despite the different muscle tone between the two conditions.^10^^,^^11^ The femoral growth plate “lateral tilt” may represent a more unifying cause for the development of hip displacement (HD), leading to progressive coxa valga with acetabular dysplasia secondary to lateral pressure from the femoral head. Early intervention strategies aimed at early modulation of proximal femoral growth plate may play a role in preventing or treat HD. One such approach is the microinvasive early proximal femoral-guided growth via the surgical technique of Hip Screw Hemi-epiphysiodesis (HSHE) that has the potential to dynamically correct proximal deformities during growth as reported in children with CP.^15^^,^^16^ There has been increasing interest and preliminary findings supporting the use of HSHE in selected group of patients with SMA.^10^^,^^11^

In addition to the mechanical aspects, hip pain can be an important issue in patients with SMA and proactive measurements and awareness should be heightened among health care providers. Chronic pain, including hip pain has been reported in a significant portion of patients with SMA, particularly in type 2 SMA (49%) in comparison to SMA type 1 (12%).^16^

The approach to hip pathology remains controversial among clinicians worldwide, ranging from conservative strategies to aggressive surgical management. Previous studies have attempted to provide orthopaedic care recommendations for children with SMA following treatment with DMT, but significant gaps remain in understanding the disease trajectory.

A 2022 consensus from the European Neuromuscular Centre Standard of Care (ENMC) proposed a more proactive approach to treat hip instability to capitalise on the treatment's effects and allow for maximal mobility.^17^ This contrasts with the traditional approach, which tends to be more conservative due to concerns over recurrent hip dislocations after surgery and the associated surgical and anaesthetic risks in the SMA population.^18^, ^19^, ^20^

To address this evolving landscape and the lack of consensus in international clinical practice, we aimed to undertake a Delphi consensus exercise involving multidisciplinary SMA teams from all major UK paediatric centres, supported by the SMA REACH and SMA CARE Networks, the British Society for Surgery in Cerebral Palsy and British Society for Children's Orthopaedic Surgery. This is the first attempt in the UK to provide an interdisciplinary consensus statement of experts to act as formal guidelines for both prevention and management of hip problems in children with SMA in the DMTs era.

## Methods

### Study design and ethics

In September 2023, orthopaedic surgeons, paediatric neurologists and physiotherapists, involved in the care of children with neuromuscular disorders, were invited to a workshop organized by the SMA REACH UK (the national clinical and research SMA network in the UK). The focus was musculoskeletal issues in children with SMA, particularly HD prevention and management. The meeting aimed to review current practice and address new challenges in orthopaedic care for patients with SMA.

Following the workshop, a core working group (MK, MV, JS, FN, GB, JL, and PT), including a patient representative, was formed from two major UK SMA multidisciplinary centres—Great Ormond Street Hospital and Evelina London Children's Hospital. These centres manage a high volume of patients with SMA and have established multidisciplinary expertise in neuromuscular and orthopaedic care. The working group was formed based on clinical experience, long-standing involvement in SMA multidisciplinary services, and expressed interest and motivation to lead the development of a national consensus on hip displacement management. The group was responsible for coordinating the Delphi process, drafting the initial statements, and incorporating feedback between rounds.

While a systematic literature review was not a primary aim of this Delphi consensus exercise, we conducted a focused literature review to ensure relevant evidence was considered. We conducted a systematic literature search with no language restrictions on PubMed, from database inception to September 2025, including studies of all designs. Search terms included “hip surgery in SMA,” “hip displacement in SMA,” “pain hips SMA,” and “orthopaedic surgery in SMA.” Twelve relevant papers were identified through this process and have been included as references.

All 23 paediatric neuromuscular centres part of SMA REACH UK were invited to participate, each including at least three specialists (orthopaedic surgeon, paediatric neurologist, and physiotherapist), ensuring a wide and diverse geographical representation of the country.

This Delphi consensus study involved two rounds: Round 1 (online questionnaire) and Round 2 (hybrid live event: online and in-person participation).

No ethical approval was required for this study, as it was not a research study and it did not involve the collection of new patients. No original data was collected. It was an expert consultation aimed at quality improvement. All participants in the Delphi process agreed to voluntarily take part and to be listed as contributors of this publication.

### Delphi process: round 1

In Round 1, a questionnaire including 16 statements was designed by the core working group. The 16 statements were developed through structured discussion within the core working group following the SMA REACH UK workshop, informed by key clinical questions, areas of variation in current practice, and priorities identified during multidisciplinary and patient representative discussions. The questionnaire was distributed from August to September 2024 via an online survey. It was distributed through members of the SMA REACH Network and members of the British Society for Children's Orthopaedic Surgery (BSCOS) and British Society for Surgery in Cerebral Palsy (BSSCP). A representative member from the advocacy group SMA UK was also invited to participate.

Participants scored each statement using the Grading of Recommendations Assessment, Development and Evaluation (GRADE) scale, which ranges from 1 to 9 (1–3 = not important, 4–6 = important but not critical, and 7–9 = critical for inclusion).^21^ The option of not answering was given if the respondent felt that the topic of the statement was outside of their expertise. A free-text response box was available to provide feedback or propose new statements.

Responses were anonymous. Statements scoring between 7 and 9 by ≥ 75% of the participants were defined as “critical for inclusion” and therefore approved. Statements scoring between 1 and 3 by ≥ 25% of the participants were defined as “not important for inclusion” and excluded. Statements scoring in the 4–6 range were defined as “important but not critical for inclusion” and later revised by the core working group based on feedback and carried forward to Round 2.

### Delphi process: round 2

A modified questionnaire containing the remaining seven statements requiring further discussion was created by the core working group, with no changes made to the wording of these statements.

In November 2024, participants were invited to an in-person meeting to facilitate discussion. To ensure consistent and updated knowledge among participants, subspecialised lectures were given including current evidence covering surgical procedures, hip surveillance, radiographic measurements, natural history of musculoskeletal problems and hip displacement in SMA, and comparison to available data on CP. SMA UK advocacy group gathered feedback from families, which was shared during the session.

Live voting was conducted using Mentimeter (Interactive presentation software; Stockholm, Sweden: available at: https://www.mentimeter.com), an interactive tool that support real-time anonymous voting and writing feedback. A maximum of three representatives from each neuromuscular centre was invited to participate in the voting. Consensus was achieved when individual statements achieved a score of 7–9, voted upon by ≥75% of all participants. Responses were anonymous.

Final consensus was created from the approved statements in both rounds.

### Role of the funding source

The funders had no involvement in study design, data collection, data analyses, data interpretation, or the writing of the report.

## Results

**Fig. 1:**
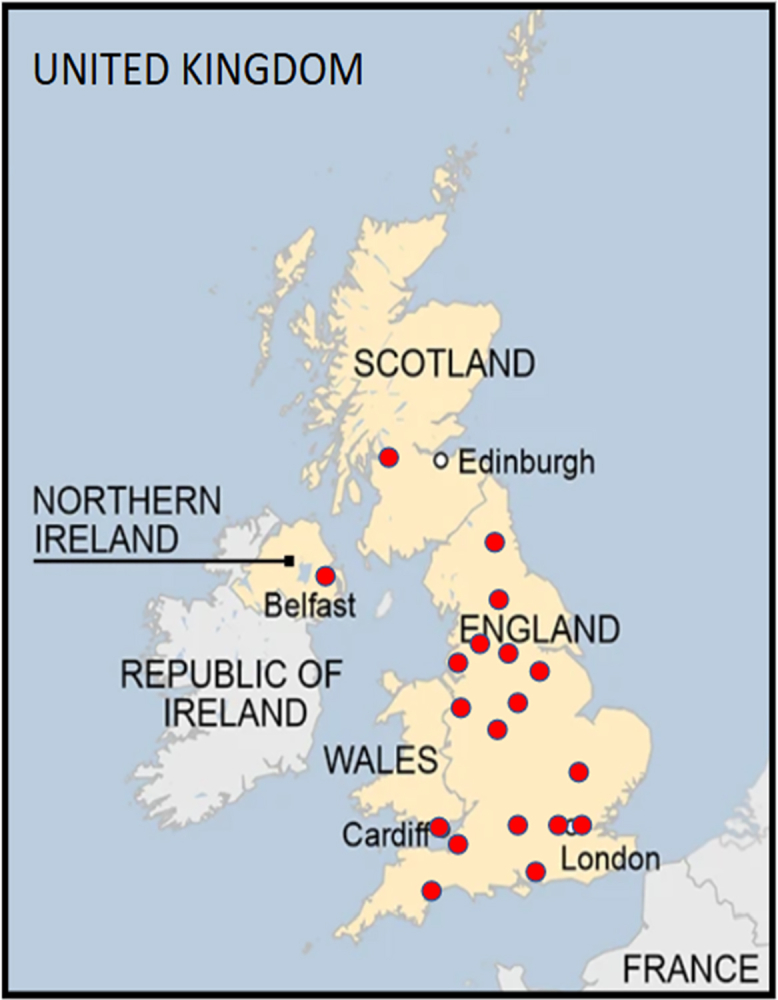
Geographical Distribution of participating UK Paediatric Institutions.

Of the 23 paediatric neuromuscular centres in the UK that were invited to participate, 19 centres agreed to participate in this Delphi consensus process (Fig. 1).

Round 1 included 16 statements within the online questionnaire, collated by the core working group, that covered six key topic areas: hip displacement prevention and surveillance, multidisciplinary care and data recording, principles and indications for surgical intervention, management of painful hip displacement, perioperative management and management of associated musculoskeletal conditions.

Between August and September 2024, 45 of 69 invited participants responded to the Round 1 questionnaire (response rate 65%). Respondents had at least 5 years’ relevant experience in the field and comprised 12 paediatric neurologists, 15 orthopaedic surgeons, and 17 neuromuscular or orthopaedic physiotherapists from 19 paediatric neuromuscular centres. (representing 82% of all centres), along with one patient representative (Table 1). Six statements achieved consensus in Round 1, addressing the following topics: specialist multidisciplinary team assessment in tertiary centres, systematic recording of orthopaedic and radiological data within a national SMA registry, the need to develop evidence-based guidelines for hip surgical interventions through audit and research activity, consideration of treatment for painful hip displacement, the selective role of major hip surgery following multidisciplinary assessment, and the management of associated muscle or joint contractures on an individualised basis. Three statements were rejected and seven were included for Round 2 discussion (Table 2).

**Table 1:** Composition of the UK SMA hip consensus group.

First name initial	Surnames	Profession/Job Title	City	Years of experience
A	Fishlock	Consultant Paediatric Orthopaedic Surgeon	Leeds	5+ years
A	Manzur	Consultant Paediatric Neurologist	London	5+ years
A	Cosgrove	Consultant Paediatric Orthopaedic Surgeon	Belfast	5+ years
A	Hipwell	Paediatric Physiotherapist	London	5+ years
A	Palanivel	Consultant Paediatric Orthopaedic Surgeon	Manchester	5+ years
A	Majumdar	Consultant Paediatric Neurologist	Bristol	5+ years
A	Childs	Consultant Paediatric Neurologist	Leeds	5+ years
B	Carsi	Consultant Paediatric Orthopaedic Surgeon	Stoke-on-Trent	5+ years
C	Edwards	Consultant Paediatric Orthopaedic Surgeon	Plymouth	5+ years
C	Frimpong-Ansah	Paediatric Physiotherapist	Plymouth	5+ years
C	Murnaghan	Consultant Paediatric Orthopaedic Surgeon	Glasgow	5+ years
C	Carpenter	Consultant Paediatric Orthopaedic Surgeon	Cardiff	5+ years
D	Rad	Consultant Paediatric Orthopaedic Surgeon	Southampton	5+ years
D	Baskaran	Consultant Paediatric Neurologist	Leicester	5+ years
E	Wraige	Consultant Paediatric Neurologist	London	5+ years
E	O'Reilly	Paediatric Physiotherapist	London	5+ years
E	Dowling	Paediatric Physiotherapist	Nottingham	5+ years
F	Henderson	Paediatric Physiotherapist	Oxford	5+ years
F	Norman-Taylor	Consultant Paediatric Orthopaedic Surgeon	London	5+ years
F	Henderson	Paediatric Physiotherapist	Oxford	5+ years
G	Baranello	Consultant Paediatric Neurologist	London	5+ years
G	nic fhirléinn	Paediatric Physiotherapist	Belfast	5+ years
I	Horrocks	Consultant Paediatric Neurologist	Glasgow	5+ years
I	Hughes	Consultant Paediatric Neurologist	Manchester	5+ years
I	Shah	Consultant Paediatric Orthopaedic Surgeon	Manchester	5+ years
J	Taylor	Paediatric Physiotherapist	Cambridge	5+ years
J	Widnall	Consultant Paediatric Orthopaedic Surgeon	Liverpool	5+ years
J	Sheehan	Paediatric Physiotherapist	London	5+ years
J	Turner	Consultant Paediatric Orthopaedic Surgeon	Bristol	5+ years
J	Cashman	Consultant Paediatric Orthopaedic Surgeon	Sheffield	5+ years
J	Longatto	Paediatric Physiotherapist	London	5+ years
K	White	Paediatric Physiotherapist	Sheffield	5+ years
L	Pallant	Paediatric Physiotherapist	Leeds	5+ years
M	Vanegas	Consultant Paediatric Neurologist	London	5+ years
M	Scoto	Consultant Paediatric Neurologist	London	5+ years
M	Main	Paediatric Physiotherapist	London	5+ years
M	Illingworth	Consultant Paediatric Neurologist	Southampton	5+ years
M	Mifsud	Consultant Paediatric Orthopaedic Surgeon	Oxford	5+ years
M	Kokkinakis	Consultant Paediatric Orthopaedic Surgeon	London	5+ years
M	Geary	Paediatric Physiotherapist	Southampton	5+ years
M	Ong	Consultant Paediatric Neurologist	Sheffield	5+ years
N	Taylor	Paediatric Physiotherapist	Bristol	5+ years
N	Emery	Paediatric Physiotherapist	Oswestry	5+ years
N	Kiely	Consultant Paediatric Orthopaedic Surgeon	Oswestry	5+ years
P	Thorman	Head of Advocacy and Community	London	5+ years
R	Buckingham	Consultant Paediatric Orthopaedic Surgeon	Oxford	5+ years
R	Keetley	Paediatric Physiotherapist	Nottingham	5+ years
R	Madhu	Consultant Paediatric Neurologist	Liverpool	5+ years
R	Muni Lofra	Paediatric Physiotherapist	Newcastle	5+ years
R	Rabb	Paediatric Physiotherapist	Birmingham	5+ years
S	Gregson	Paediatric Physiotherapist	Liverpool	5+ years
S	Ramdas	Consultant Paediatric Neurologist	Oxford	5+ years
S	Warner	Paediatric Physiotherapist	Manchester	5+ years
S	Annamalai	Consultant Paediatric Orthopaedic Surgeon	Leicester	5+ years
S	McKenzie	Paediatric Physiotherapist	Glasgow	5+ years
T	Willis	Consultant Paediatric Neurologist	Oswestry	5+ years
W	Atherton	Consultant Paediatric Orthopaedic Surgeon	Bristol	5+ years
Z	Lambat	Paediatric Physiotherapist	Leicester	5+ years

**Table 2:** UK consensus statements on hip displacement management in spinal muscular atrophy in the era of disease-modifying therapies.

UK Consensus Statements on Hip Displacement Management in Spinal Muscular Atrophy in the Era of Disease-Modifying Therapies	n/N (%) in agreement
1. An effort should be made to prevent hip dislocation in children with SMA; this can be more relevant when expected to achieve higher motor abilities (i.e. assisted or unassisted standing/walking).	26/33 (78%)
2. Annual radiographic hip surveillance, starting ideally between 6 and 18 months, is recommended for children with SMA.	31/34 (92%)
3. Radiographic hip surveillance can be adjusted to minimise radiation exposure, as we learn more about the natural history of hip displacement in SMA and its prevention and treatment.	33/34 (98%)
4. Radiographic signs to check on Hip X-rays: Hip displacement (Reimer's Migration Percentage), acetabular dysplasia (Acetabular Index) and Head-Shaft Angle.	13/13 (100%)
5. Multidisciplinary Team Assessment: Children with SMA should undergo assessment in a tertiary referral setting, by a multidisciplinary team (MDT) consisting of an orthopaedic surgeon, a neurologist and a physiotherapist.	38/45 (84%)
6. Inclusion in Registry: All relevant clinical information regarding orthopaedic and radiological assessments should be documented and included	39/45 (87%)
7. SMA is not a contraindication to hip surgery per se: A case by case risk/benefit approach should be in place.	33/33 (100%)
8. Guidelines for Surgical Interventions: An effort should be made to develop evidence-based guidelines around Hip Surgical Interventions in children with SMA, and this should be conducted as part of audit strategies and research activity.	43/45 (95%)
9. Guided Growth hip surgery may be considered for selected patients following regional MDT case by case discussion.	29/34 (85%)
10. Treatment of Painful Displacements: Children with painful displaced hips should be considered for treatment, unless contraindicated.	45/45 (100%)
11. Steroid hip injections can be considered in the management of hip pain for both diagnosis and treatment.	30/32 (93%)
12. Indication for Major Surgery: Major hip surgery may be considered as a treatment option for selected children with painful displaced hips following MDT assessment.	35/45 (78%)
13. Management of Concomitant Contractures: Surgically treating any concomitant muscle or joint contractures should be considered on a case by case basis particularly for those who are achieving higher motor milestones (stander with or without AFOs/KAFOs and use of hands, or ambulant).	38/45 (84%)

Abbreviations: SMA = spinal muscular atrophy; MDT = multidisciplinary team; AFO = ankle–foot orthosis; KAFO = knee–ankle–foot orthosis.

In November 2024, 34 of 69 invited participants (response rate 49%) joined Round 2 for a live discussion, seven statements were included and four of them modified during the meeting. Statements requiring further discussion in Round 2 focused on hip displacement prevention in relation to motor function, the timing and frequency of radiographic hip surveillance and strategies to minimise radiation exposure, key radiographic parameters for assessment, the principle that spinal muscular atrophy is not a contraindication to hip surgery, and the role of minimally invasive prophylactic procedures and steroid hip injections in selected patients. These statements were discussed in detail during Round 2, modified where appropriate based on multidisciplinary and patient feedback, and all subsequently reached consensus and were approved.

The SMA UK advocacy group gathered feedback from families and individuals living with SMA through a patient perspective survey conducted as part of the SMA Care programme. The survey received responses from 51 carers and 20 individuals living with SMA and was distributed via SMA UK social media channels and community WhatsApp networks. In addition, SMA UK facilitated a follow-up virtual discussion on hip management with six parent volunteers whose children had either undergone hip surgery or were actively discussing surgical options with their clinical teams. Data from the survey and the virtual discussion were synthesised and presented by SMA UK in a summary presentation during the Round 2 workshop to inform multidisciplinary discussion prior to completion of the second-round statements.

The final consensus included 13 approved statements with each statement achieving a score between 7 and 9 (by ≥75% of all participants), which satisfied the consensus definition (Table 1).

## Discussion

This is a first national initiative in the UK that attempts throughout a Delphi consensus to provide some guidance for the surveillance and management of hip displacement in symptomatic children with SMA in the era of DMTs. Senior clinicians from major UK paediatric neuromuscular centres, and with relevant involvement in the care of SMA children, participated across two rounds of consultation. Agreement was achieved on 13 statements that together outline recommendations to early detect and manage HD in the current rapidly changing clinical landscape.

There was a collective view that an effort should be made to prevent hip dislocation wherever possible. The proposed approach was to adopt a preventive strategy, which could be even more relevant in children expected to achieve higher functional abilities, like assisted or independent standing and walking. This reflects the shift from the traditional approach—where limited motor function and high surgical risk limited management options—to a contemporary context in which DMTs have redefined the expected trajectories and opened the outcomes to new therapeutic possibilities. Participants emphasised the need to have individualised decision-making by experienced multidisciplinary teams, integrating motor milestones, functional goals, family priorities, and updated evidence regarding conservative and surgical strategies.

Similar to the successful hip surveillance programmes established for children with cerebral palsy,^22^ continuous radiographic hip surveillance was considered essential for improving understanding of the new disease trajectory of hip displacement in children with SMA and its relationship with clinical symptoms and mobility limitations. The group supported annual radiographic hip surveillance, ideally beginning between 6 and 18 months of age, recognising the early onset and rapid progression of HD in SMA compared with other neuromuscular disorders. However, the consensus also acknowledged the need to minimise radiation exposure and to refine surveillance frequency as new disease trajectory data emerge.^23^ No recommendation was made regarding a specific age for stopping surveillance, consistent with the principle of individualised consideration, influenced by functional improvement, skeletal maturity, and evolving clinical needs.

Agreement was reached on a core set of radiographic indicators: Reimer's Migration Percentage (RMP), Acetabular Index (AI), and Head–Shaft Angle (HSA). Only participants with expertise in interpreting hip x-rays voted on this statement. It was agreed that an effort should be made to harmonise data collection to build a robust dataset capable of informing future evidence-based recommendations.

**Fig. 2:**
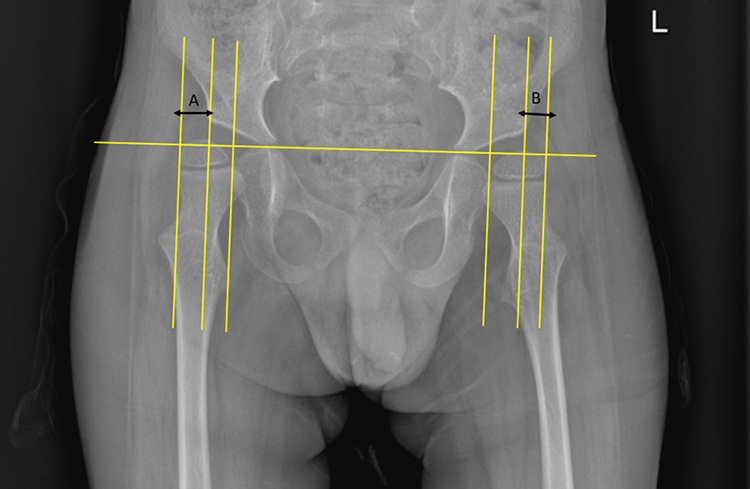
Reimer's migration measurement for right hip(A) and left hip(B).

The Reimer's Migration Percentage is the recognised radiographic measurement in hip surveillance in children with CP^23^ (Fig. 2). RMP is the most important measurement on an Antero-Posterior (AP) hip radiograph obtained for hip surveillance describing the percentage of the ossified femoral head (ossific nucleus) which lies outside (lateral) of the osseous acetabular socket. It is defined as the percentage of the ratio of the uncovered ossific nucleus (outside the acetabulum) to its total diameter. Migration percentages equal or greater than 30% are associated with an increased risk of progressive hip displacement.^23^

**Fig. 3:**
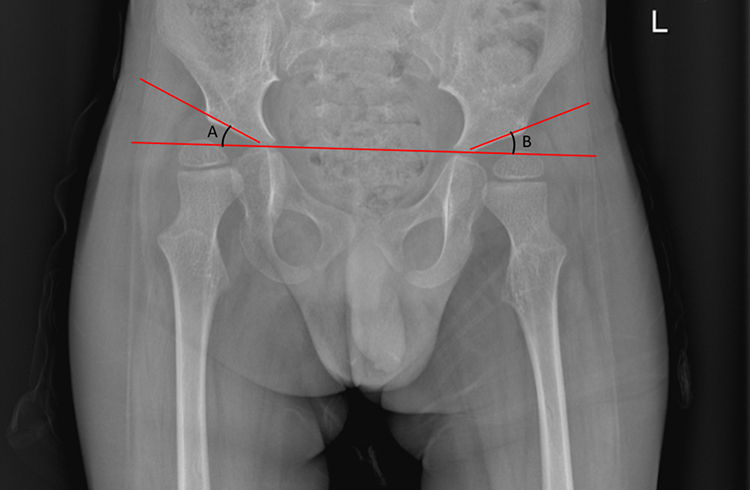
Acetabular Index measurement for right hip(A) and left hip(B).

Acetabular Index measurement will provide useful information on the disease trajectory and prevention and management of acetabular dysplasia^24^ (Fig. 3). Acetabular Index is a widely used radiographic parameter on an AP hip radiograph to describe dysplasia of the acetabulum and not specifically in children with CP. It is defined as the angle formed between the iliac part of the acetabulum and the horizontal and describes how shallow the acetabular socket is and consequently if the femoral head is pre-disposed to lateral displacement. Normal values of Acetabular Index range per age and gender.

**Fig. 4:**
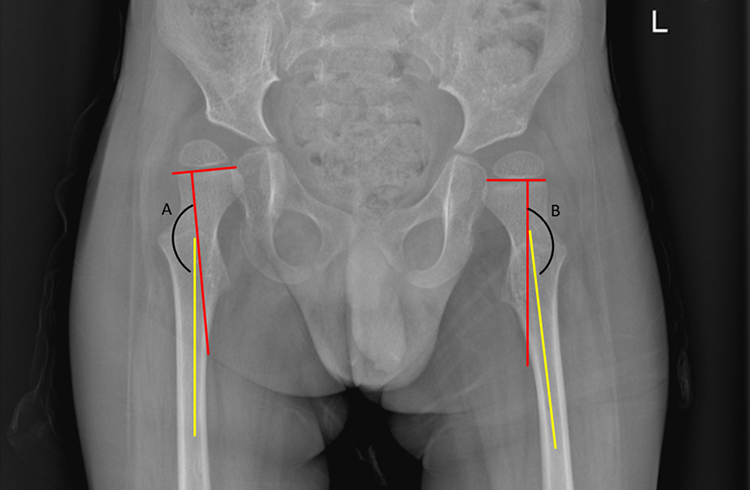
Head-Shaft Angle measurement for right hip(A) and left hip(B).

The inclusion of Head–Shaft Angle, traditionally associated with cerebral palsy, reflects growing interest in identifying early markers of risk in SMA, particularly as phenotypes evolve in the DMT era^25^ (Fig. 4). HSA is a key measurement on AP hip radiographs to describe the alignment of the femoral head relatively on its femoral shaft. The degree of outward tilt (i.e. coxa valga as defined by HAS measurements >160°) is strongly associated with greater risk of hip displacement.

Multidisciplinary team (MDT) approach to manage musculoskeletal problems in children with SMA is essential to maximise standards of care.^26^^,^^27^ The consensus reinforced the importance of MDT-led assessments in tertiary referral settings, supported by close collaboration with local teams. It was acknowledged that the provisions of MDT assessments depend on available resources and could be variable among different centres. However, it was recommended that cases involving surgical considerations should undergo tertiary-level discussion to ensure coordinated and expert-driven decision-making.

A primary topic of discussion concerned data collection and integration into the national registry (SMA REACH UK registry). Participants emphasised the critical role of disease registries in generating real-world evidence, particularly for rare conditions where prospective studies are difficult to conduct.^28^ Key aspects that were identified included: motor function scales, segmental muscle strength, range of movements/contractures, quality of life, pain assessment, surgical intervention outcomes and post-operative recovery, in addition to the set of radiographic indicators mentioned above. However, it was acknowledged that this represents only an initial overview of the principal domains. To further refine and characterise these elements, including a more comprehensive approach to conservative non-surgical management and longitudinal clinical outcomes to collect, additional working group meetings have already been scheduled.

Regarding surgical interventions, there was strong consensus (43/45 votes, 95%) that evidence-based guidelines should be developed for hip surgery in children with symptomatic SMA, ideally integrated within audit strategies and research activities. Although current evidence supports consideration of surgical intervention for both prevention and management of hip displacement,^13^ it remains important to generate short-, medium-, and long-term data on the effectiveness of hip surgery in this population, as well as to evaluate the potential outcomes of non-operative management strategies.

The group concluded that SMA is not a contraindication to hip surgery per se, countering long-standing scepticism regarding surgical intervention in this patient population. This reflects a shift in the mindset and practice driven by DMTs and emerging evidence suggesting potential benefit of orthopaedic surgery. Although gaps remain in the current knowledge of this evolving phenotype, participants endorsed a more proactive stance toward surgical options.

Possible surgical interventions that were discussed included guided growth hip surgery and hip reconstruction surgery. The statement was discussed in Round 2 following explanation of the procedure and its potential benefits in both prevention and management of HD in children with SMA.^10^^,^^11^ The minimally invasive nature of guided growth makes this a desirable option for children with SMA, who despite the DMTs introduction, face complex co-morbidities and increased peri-operative risks. Open discussion was facilitated and there was majority agreement about considering minimally invasive surgical options for selected patients, while acknowledging the lack of current evidence and the need to collect long-term data, ideally within research setting.

At present, long-term outcomes for guided growth surgery remain limited in cerebral palsy, where the procedure has been increasingly adopted in the last years as the proactive, minimally invasive technique reducing peri-operative morbidity and allowing rapid post-operative recovery and fast return to preoperative function. Ongoing European multicentre prospective studies (including two randomised controlled trials from the Netherlands and Spain and one multicentre prospective observation study from the UK) are expected to provide further insight on the efficacy and durability of this intervention in cerebral palsy, with potential implications for its applications in SMA.^29^, ^30^, ^31^

Hip reconstruction surgery in the pre-DMTs era was reported to be associated with increased risk of complications such as re-dislocation mainly due to the hypotonic and weak muscles which fail to retain the reduced femoral head in the acetabular socket, which is a recognised problem when considering hip reconstructive procedures to similar hypotonic children with cerebral palsy or presenting in many genetic chromosomal abnormalities.^32^

In the post-DMTs era however, and in particular when DMTs are started in the first few days/weeks of life, improved muscle tone and strength, as opposed to the severe hypotonia and muscle weakness of untreated patients, could in principle represent and advantage to reduce the high risk of failure after hip reconstruction. The amount of hypotonia and weakness will also determine the speed and potential to adequate post-operative recovery and return to pre-operative mobility and function after major orthopaedic surgery. However, it was acknowledged that more longitudinal long-term data will be needed to define the clinical and functional benefits of hip surgery in symptomatic SMA children treated with DMTs.

Management of painful hip displacement was a further area of clear agreement. Chronic pain in children is a cause of functional disability.^33^ It is an essential duty of health care professionals looking after children with neurological problems, to make every possible effort to manage pain in an effective way.

Both non-operative and/or operative treatment methods should be considered when facing the scenario of a child with SMA complaining of painful hip joints where radiographs show HD or complete dislocation. HD is defined as a Reimer's migration greater than 30% while hip dislocation is determined as a Reimer's migration greater than 80%.^34^

There was consensus on the potential role of intra-articular corticosteroid injections as a diagnostic tool to localize pain, identify patients most likely to benefit from surgical reconstruction, and, in some cases, potentially avoid the need for hip surgery.^35^ However, data on long-term safety and optimal dosing or frequency remain limited.^36^^,^^37^

Hip reconstructive surgery is an appropriate treatment option for children with painful displaced hips when non-operative management or minimally invasive strategies (e.g., intra-articular corticosteroid injections) fail to provide adequate pain relief. Although our overall approach prioritises proactive and preventative strategies, the group acknowledged that many symptomatic chronic patients with SMA are likely to present with painful hips.^16^ it was acknowledged that further surgical options including hip salvage procedures or hip replacement surgery should be considered when making decisions on the reactive management of painful dislocated hips of teenagers or young adults as it is the case in the cerebral palsy population.^38^^,^^39^

Consequently, there was consensus that guidance on the reactive management of painful displaced hips should be included. Such procedures should be planned and undertaken within an interdisciplinary framework to ensure fully informed decision-making and optimal preoperative preparation, thereby reducing perioperative risk and improving surgical outcomes.

Similarly, addressing concomitant contractures—particularly in children achieving higher motor milestones—was considered essential for maintaining function, with both conservative and surgical approaches deemed appropriate.^9^ Further discussion and guidance on non-operative strategies will follow and remain an ongoing focus of the working group.

This work has some limitations. These consensus recommendations are primarily based on expert opinion rather than prospective evidence, reflecting gaps in long-term data on surgical and non-operative outcomes. Detailed guidance on non-operative strategies remains pending and is part of the ongoing work of the group. Another limitation is that only informal patient and caregiver perspectives were incorporated, as patient representative provided initial feedback to the working group, and took part in the final round of consensus. Finally, the rapidly evolving SMA landscape, including emerging phenotypes and new therapeutic options, may require continuous updates to maintain the clinical relevance of these recommendations.

In conclusion, this is an extensive multicentre, multidisciplinary effort to provide surgical guidelines for managing hip problems in children with SMA in the era of DMTs. There is limited data about natural history of hip displacement in the new phenotypes of SMA, and a Delphi consensus from an interdisciplinary and diversified group of experts offers valuable guidance. However, further multicentre research is needed to better understand this changing landscape and refine the current guidance.

## Contributors

MV, GB, FN and MK conceptualised the consensus process. All authors contributed to the development of the consensus statements. MV, GB and MK accessed and verified the underlying data. MV, GB and MK drafted the manuscript. All authors critically revised the article and approved the submitted version.

## Data sharing statement

Aggregated Delphi survey data supporting the findings of this study are included in the article and its Supplementary Materials. No individual-level data are publicly available.

## Editor note

Evidence-based guidance for orthopaedic management in Spinal Muscular Atrophy remains limited in several key areas. This Delphi consensus study brings together multidisciplinary expertise to develop practical recommendations, providing a transparent and reproducible framework to inform clinical practice and future research.

## Declaration of interests

This consensus process received funding from Novartis, Roche, and Biogen that covered the cost of the in-person meeting in London, November 2024. GB reports grants or contracts from Sarepta, Roche, Novartis, Pfizer, Italfarmaco, and Santhera to University College London; consulting fees from Sarepta, Entrada Therapeutics, Pfizer, Biogen, Roche, and Novartis; support for attending meetings or travel from Roche and Novartis; and receipt of materials or services from Roche to his institution. None of the above were related to this publication. MV reports consulting fees from Roche and support for attending meetings or travel from Roche and Novartis. None of the above were related to this publication. MK reports support for attending meetings or travel from Roche. None of the above were related to this publication. All other authors declare no competing interests.

## Appendix A Supplementary data

UK SMA Hip Consensus Group
