# Supplementary material for: Hip displacement management in spinal muscular atrophy in the era of disease modifying therapies: a Delphi consensus study in the UK
Source: eClinicalMedicine. 2026 Apr 18;95:103872. doi: 10.1016/j.eclinm.2026.103872 (PMC13098339; doi:10.1016/j.eclinm.2026.103872)
Supplement: UK SMA Hip Consensus Group [file mmc1.docx]

**UK SMA Hip Consensus Group**

*Evelina London Children’s Hospital, Guy’s and St Thomas’ NHS Foundation Trust, London: M Kokkinakis, M Vanegas, J Sheehan, E Wraige. Great Ormond Street Hospital for Children NHS Foundation Trust, London: G Baranello, F Norman-Taylor, A Manzur, M Scoto, A Hipwell, E O’Reilly, M Main J Longatto. John Walton Muscular Dystrophy Research Centre, Newcastle NHS Trust: R Muni Lofra. Oxford University Hospitals NHS Foundation Trust / Oxford Muscle Service: M Mifsud, R Buckingham, F Henderson, S Ramdas. Southampton Children’s Hospital, University Hospital Southampton NHS Foundation Trust: M Illingworth, M Geary, Royal Manchester Children’s Hospital, Manchester: I Hughes, I Shah, S Warner, A Palanivel RJAH Orthopaedic Hospital NHS Foundation Trust, Oswestry: N Emery, N Kiely, T Willis. Sheffield Children’s Hospital NHS Foundation Trust: J Cashman, M Ong, K White. Bristol Royal Hospital for Children, University Hospitals Bristol NHS Foundation Trust: J Turner, N Taylor, A Majumdar, W Atherton. University Hospitals Plymouth NHS Trust: C Edwards, C Frimpong-Ansah. Royal Hospital for Children, NHS Greater Glasgow and Clyde: I Horrocks, C Murnaghan, S McKenzie. Nottingham Children’s Hospital, Nottingham University Hospitals: R Keetley, E Dowling. Leeds General Infirmary, Leeds Teaching Hospitals NHS Trust: L Pallant, A Fishlock, A Childs. Alder Hey Children’s NHS Foundation Trust, Liverpool: J Widnall, R Madhu, S Gregson. Leicester Royal Infirmary / Leicester Children’s Hospital: D Baskaran, Z Lambat, S Annamalai. Cambridge University Hospitals NHS Foundation Trust (Addenbrooke’s Hospital): J Taylor. Cardiff and Vale University Health Board: C Carpenter. Birmingham Women’s and Children’s NHS Foundation Trust: R Rabb. Belfast Health and Social Care Trust: A Cosgrove, G Nicfhirleinn. SMA UK: P Thorman. Royal Stoke University Hospital: M Carsi.*
